# The cost of recreation: how hiking could be making birds sick

**DOI:** 10.1093/conphys/coaa058

**Published:** 2020-08-04

**Authors:** Bridie J M Allan

**Affiliations:** Department of Marine Science, University of Otago, 310 Castle Street, PO Box 56, Dunedin 9054, New Zealand

Humans are inextricably linked to the environmental landscape within which our daily lives unfold. We depend on our environment not only for essential services, such as water and food, but increasingly for recreation. However, as human populations continue to grow and propagate across landscapes, our presence is having a marked effect on wildlife; even low-impact activities, such as hiking, can stress animals.

Is it possible that, despite some of our best efforts to be respectful to wild populations, we are inadvertently damaging them? Could the seemingly benign activity of bird watching actually be harming the birds we love? According to a team of researchers in France studying adult Eurasian blue and great tits, the answer is yes—at least for their nestlings.

Blue and great tits are small passerine (perching) birds. Widespread throughout Europe and western Asia, they are easily recognizable by their blue and yellow plumage. However, like most birds, they get stressed out when disturbed, especially by human presence. This led Yves Bötsch and his team to ask whether recreational activities, such as hiking during the bird breeding season, would stress mother birds and whether this would affect maternal transfer of antibodies in the nestlings of blue and great tits.

Why antibodies? Well, antibodies are really important for immunity and coping with infection. For birds, immunity develops as nestlings mature. This means that newly hatched nestlings rely entirely on antibodies received from the mother during early development to buy them time while they are making their own.

Bötsch and his team hiked through the Forêt Domaniale de Chaux in France carrying a loudspeaker broadcasting human conversations to reproduce normal hiking conversation across two consecutive breeding seasons. Following each ‘exposure’ period, 6-day-old nestlings were weighed and blood samples were taken to measure maternally transmitted antibodies. Nestlings were weighed again 9 days later to track their growth rates.

Interestingly, the team found that if the mothers were exposed to human activity, the number of antibodies the mother passed on to their nestlings decreased and this had a negative effect on nestling growth and survival. So, why were there less antibodies in the nestlings of disturbed mothers? The team suggests that the most likely cause is increased corticosterone, which is a hormone released during stressful conditions. Corticosterone prepares the body for the all-important fight-or-flight response. However, it can also suppress the immune system, which could be one of the reasons why antibodies decreased.

Why does this matter? Bötsch and his team point out that nestlings could hatch without the full supply of tools needed to fight infection. If disturbances continue through the entire breeding season, these problems are further magnified. For example, in addition to reduced antibodies, nestlings from stressed mothers will also face shorter incubation times, which means smaller, more vulnerable hatchlings with slower growth rates.

So, how do we minimize this risk? Bötsch and his team suggest closing areas to hikers during breeding seasons and focussing on areas with vulnerable bird populations. The voices we use to convey excitement about observations in the natural world also have the power to damage the things we love to observe. So, how do we communicate when in nature? We can certainly whisper and lower our voices, and we might even see more wildlife!

Illustrations: Erin Walsh, ewalsh.sci@gmail.com


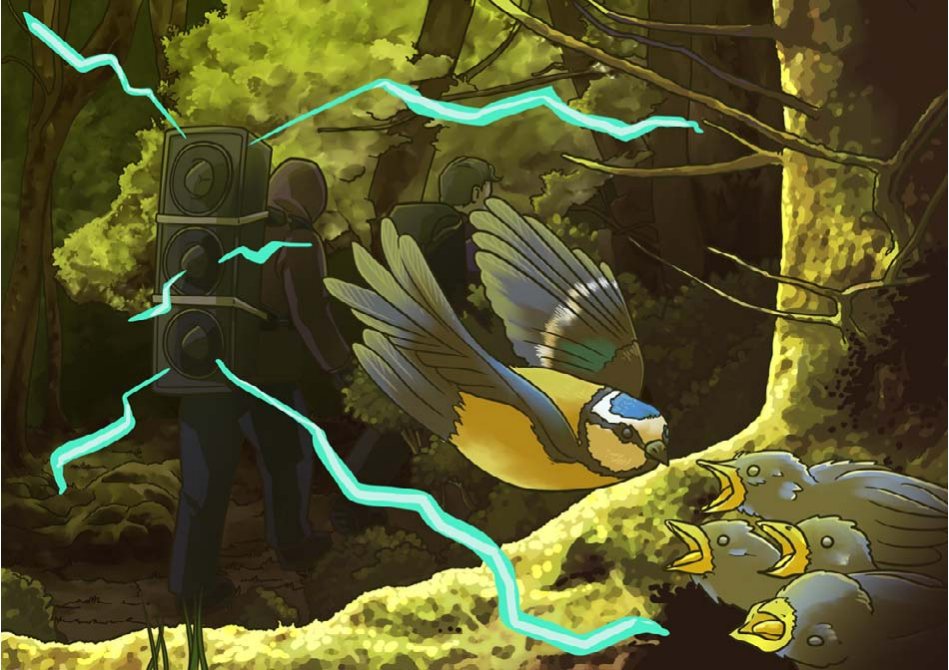


Editor: Jodie L. Rummer
